# Evolving fitness and immune escape: a retrospective analysis of SARS-CoV-2 spike protein (2020-2024) using protein language model

**DOI:** 10.3389/fimmu.2025.1576414

**Published:** 2025-06-18

**Authors:** Sihua Peng, Leke Lyu, Ludy Registre Carmola, Sachin Subedi, M. H. M. Mubassir, Mohamed A. Bakheet, Justin Bahl

**Affiliations:** ^1^ Center for Ecology of Infectious Diseases, University of Georgia, Athens, GA, United States; ^2^ Department of Infectious Diseases, University of Georgia, Athens, GA, United States; ^3^ Institute of Bioinformatics, University of Georgia, Athens, GA, United States; ^4^ Department of Epidemiology and Biostatistics, University of Georgia, Athens, GA, United States

**Keywords:** SARS-CoV-2, spike protein, protein language models, protein fitness, immune escape, retrospective analysis

## Abstract

**Introduction:**

The COVID-19 pandemic posed global health challenges. Understanding SARS-CoV-2’s evolutionary dynamics, especially fitness and immune escape, is vital for public health. This study uses protein language models to assess how genetic variations affect viral adaptability and immunity.

**Methods:**

We applied the CoVFit model to predict Fitness and Immune Escape Index (IEI), validated by a null model based on neutral evolution. We analyzed 2,504,278 SARS-CoV-2 spike sequences, including 160,892 variants, tracking evolution from 2020 to May 2024, comparing real and random mutants’ Fitness and IEI.

**Results:**

Our analysis revealed an increase in Fitness (mean rising from 0.227 in 2020 to 0.930 in 2024) and IEI (mean increasing from 0.171 to 0.555) for North American samples. Globally, the comparison of Fitness and IEI between real and random mutants (generated by the null model) revealed statistically significant differences (real mutant Fitness 0.3849 vs. random mutant 0.2046, p < 0.001, KS test; real mutant IEI 0.2894 vs. random mutant 0.1895, p < 0.001, KS test), indicating strong selective pressure; the JN.1 lineage dominated (94% of sequences by April 2024), underscoring its evolutionary advantage.

**Conclusions:**

CoVFit offers key insights into SARS-CoV-2 evolution, aiding vaccine design. Persistent viral adaptation despite interventions highlights the need for surveillance and adaptive strategies using tools like CoVFit for preparedness.

## 1 Introduction

The COVID-19 pandemic, caused by SARS-CoV-2, has posed an unprecedented global challenge (1). SARS-CoV-2, a positive-sense single-stranded RNA virus, exhibits a high mutation rate that has led to the emergence of numerous variants, significantly enhancing its transmissibility and immune escape capabilities (2–4). Although vaccination has reduced severe disease rates, the virus’s ongoing evolution continues to challenge vaccine strategies (5, 6). The SARS-CoV-2 spike (S) protein is a critical determinant of its ability to infect human hosts, facilitating viral entry by binding to ACE2 receptors (7), and serves as the primary target for most vaccines due to its pivotal role in mediating viral entry and eliciting neutralizing antibody responses (8–10). The evolutionary trajectory of the virus was profoundly shaped by factors such as immune evasion, environmental pressures, and genetic alterations (2–5). Consequently, tracking the fitness and immune escape trends of the S protein is essential for developing novel vaccines and therapeutic strategies. However, traditional analytical approaches struggled to comprehensively capture its long-term dynamics, necessitating advanced computational tools to elucidate the patterns of viral evolution.

Protein language models (PLMs) offer a novel perspective for studying viral evolution by treating amino acid sequences as analogous to sentences, leveraging large-scale sequence data to capture the biochemical properties of proteins (11). The advent of transformer architectures has marked a significant advancement in predictive capabilities (12). For instance, Rao et al. pioneered the application of BERT to protein prediction with the TAPE model (13), Elnaggar et al. expanded its utility across diverse protein families with ProtTrans (14), and Rives et al. enhanced downstream task performance with ESM-1b, trained on the UniProt database (15). Building on these advances, Lin et al. developed ESM-2, a model with 1.5 billion parameters capable of directly inferring protein structures (16), while Ito et al. fine-tuned ESM-2 using genotype-fitness data and high-throughput deep mutational scanning (DMS) experimental data (17, 18) to create CoVFit, mapping the fitness landscape of SARS-CoV-2 (19). DMS, integrating deep sequencing with systematic mutation analysis, provides robust support for assessing the immune escape potential of the S protein (20, 21).

Recently, PLMs have gained prominence in SARS-CoV-2 evolutionary research. Ma et al. devised a deep learning model combining regularity and stochasticity to predict viral evolution, validating several highly transmissible variants, though its 5,000-epoch training may risk overfitting (22). Lamb et al. trained a model on 65 million protein sequences using ESM-2 to uncover evolutionary potential, yet it lacks domain-specific fine-tuning (23). King et al. developed the VPRE model to forecast future variants with limited data, but its reliance on 20,000 synthetic sequences may introduce artefactual features (24). Elkin et al. proposed the DNMS approach to predict novel S protein mutations, limited to single amino acid substitutions (25). Zvyagin et al. employed the GenSLM model to simulate viral dynamics, constrained by a smaller training dataset (26). While these studies demonstrate the potential of PLMs, they predominantly focus on specific variants or short-term trends and often rely on statistical models that overlook mutation interactions (27–29), failing to provide a global analysis of S protein dynamics throughout the pandemic.

Prior studies, such as Islam et al. (30) on XBB.1.5 immune escape and Hasan et al. (31) on vaccine efficacy and meteorological factors, have explored SARS-CoV-2 evolution. However, these efforts primarily focused on specific variants or short-term regional trends and did not address the long-term global dynamics of the S protein from 2020 to 2024. This study utilizes the CoVFit model, leveraging S protein sequences collected between January 1, 2024, and May 15, 2024, to retrospectively assess the fitness and immune escape trends of the S protein from 2020 to 2024. It aims to clarify how mutations have shaped viral characteristics across time and regions. This systematic analysis addresses the shortcomings of previous research, offering new scientific insights for predicting future variants and optimizing public health strategies.

## 2 Materials and methods

**Figure 1:**
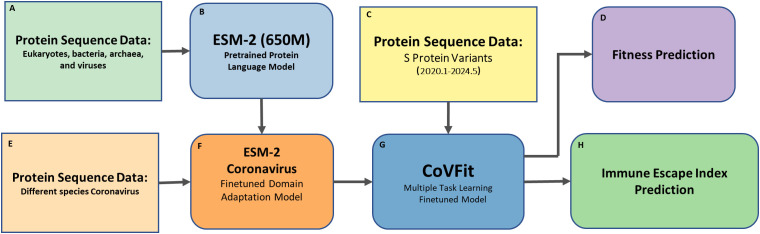
Pipeline for retrospective analysis of SARS-CoV-2 spike protein Fitness and Immune Escape (2020–2024) using CoVFit. This figure illustrates the workflow for analyzing the Fitness and immune escape index (IEI) of SARS-CoV-2 spike (S) protein variants using the CoVFit model. The pipeline first performs domain adaptation finetuning of the pretrained ESM-2 model using various coronavirus data, followed by a second finetuning step with SARS-CoV-2 data, and finally predicts the Fitness and IEI of historical SARS-CoV-2 data. The components are as follows: **(A)** Protein Sequence Data – Sequences from eukaryotes, bacteria, archaea, and viruses, used for pretraining ESM-2 (650M) model; **(B)** ESM-2 – A pretrained protein language model for learning general protein representations; **(C)** S Protein Variants – SARS-CoV-2 spike protein sequences collected from January 2020 to May 2024; **(D)** Fitness Prediction – Prediction of Fitness of S protein variants; **(E)** Protein Sequence Data (Coronavirus) – Sequences from different coronavirus species, used for domain adaptation finetuning of ESM-2; **(F)** ESM-2 (Coronavirus Finetuned) – ESM-2 model finetuned on coronavirus sequences for SARS-CoV-2-specific tasks; **(G)** CoVFit – A finetuned model supporting multiple tasks, including fitness and immune escape prediction; and **(H)** Immune Escape Index Prediction – Immune escape capability based on relative binding affinity to 1,548 monoclonal antibodies (mAbs).

The pipeline of this study is shown in **Figure 1**, with data derived from global SARS-CoV-2 S protein sequences collected from January 2020 to May 2024.

### 2.1 Data collection

A total of 2,504,278 SARS-CoV-2 S protein sequences, collected between January 1, 2020 and May 15, 2024, were downloaded from NCBI SARS-CoV-2 Data Hub. The sequences were divided into 18 segments of three-month intervals to analyze the evolutionary trajectory of SARS-CoV-2 fitness and immune evasion (https://www.ncbi.nlm.nih.gov/labs/virus/vssi/#/virus?SeqType_s=Nucleotide&VirusLineage_ss=taxid:2697049). Sequences containing more than five unidentified characters were excluded from the analysis.

### 2.2 Variant sequence identification and mutation count

In the epidemiology of SARS-CoV-2, the terms “mutation,” “variant,” and “strain” are often used interchangeably, but they have distinct scientific meanings. A protein mutation refers to an actual change in the protein sequence, such as D614G, which is the substitution of aspartic acid for glycine at position 614 of the spike protein (32). Genomes differing in sequence are typically referred to as variants. This term can be vague since two variants might differ by one or many mutations. A variant is considered a strain when it exhibits a significantly different phenotype, such as differences in antigenicity, transmissibility, or virulence (33, 34). In this study, we define “variants” as spike protein S amino acid sequences that differ by one or more mutations. Some of these variants may not show differences in antigenicity, transmissibility, or virulence, but any two variants referred to in this text do differ in their amino acid sequences, commonly termed variant sequences or simply as variants.

The downloaded S protein data over various time periods contained many duplicate sequences. Duplicate sequences within each of the 18 time periods were removed, resulting in unique amino acid sequences, henceforth referred to as “variants”, within each time period. A total of 160,892 variants were obtained (**Supplementary Table S1**). However, some samples might be duplicated across different time periods.

The positions and frequencies of mutations varied for each variant. To study the changes in mutation frequency over time, we calculated the frequency of mutations in each variant sequence. A cubic spline interpolation was used to connect the mean values of mutation counts for each time segment. The code developed to remove duplications, and count occurrence and frequency of mutations is available at https://github.com/pengsihua2023/SARS-CoV-2-Fitness-IEI.

### 2.3 Phylogenetic and molecular clock analysis

We collected global S protein sequences sampled between 2020 and 2024. A random subset of 5,000 sequences with detailed sampling dates was selected. Sequences were aligned against the Wuhan-Hu-1 reference genome using MAFFT V 7.520 (35). Phylogenetic trees were inferred using IQ-TREE V 2.2.2.6 (36)with the LG+I+G substitution model, applying the -czb flag to collapse short branches into polytomies. The patristic distances between all tips and the root of the tree were calculated using the ape package in R (37). We plotted the patristic distances against sampling dates to estimate the molecular clock rate.

### 2.4 Fitness values analysis

Ito et al. first established the ESM-2 Coronaviridae model (19) by pre-training (i.e., domain adaptation) on S protein sequences obtained from 1,506 types of coronaviruses. Then, they fine-tuned the model on genotype–fitness (Re) data and DMS data to evaluate antibody neutralization escape capabilities. Consequently, for a given S protein sequence, CoVFit can predict the Fitness value in a specific country and its ability to escape from each monoclonal antibody (mAb). Their dataset included data from 17 countries: Australia, Belgium, Brazil, Canada, Denmark, France, Germany, India, Italy, Japan, the Netherlands, South Korea, Spain, Sweden, Switzerland, the United Kingdom, and the United States. CoVFit model (https://github.com/TheSatoLab/CoVFit) was finetuned from ESM-2 protein model using two data sets: i) genotype–fitness data obtained from virus genome surveillance and ii) mutation effect data on evasion ability from humoral immunity, determined by high-throughput deep mutational scanning (DMS) experiments. Then, using a multinomial logistic model fitted to genome surveillance data from the Global Initiative on Sharing All Influenza Data (GISAID) up to November 2, 2023, the Effective Reproduction Number (Re) of each genotype in each country was estimated. Re is an epidemiological parameter used to measure the transmission potential of an infectious disease within a specific population (38, 39). As a result, 21,751 genotype-fitness data points were obtained, covering 12,914 genotypes across the 17 countries, indicating that the fitness of S protein originally derived from publicly available data set of, which (38, 39) Finally, the derived fitness data were scaled to 0–1 for the convenience of model training (38, 39).

We adopted the CoVFit protein model for this study to analyze the temporal trends in Fitness and Immune Escape Indices (IEI) and the geographical distribution of SARS-CoV-2 variants across different continents. Model files were downloaded from Zenodo (https://zenodo.org/records/10911205). We used Clustal Omega-1.2.3 (40) to perform multiple sequence alignment of the amino acid sequences and input the aligned S protein sequences into the CoVFit model to predict the Fitness value of the S protein. The average Fitness value for North America was derived as the average of the United States and Canada. For Europe, the average was derived from Germany, the United Kingdom, Switzerland, Sweden, Spain, the Netherlands, Italy, France, Denmark, and Belgium. The average of South Korea, Japan, and India represent was used for Asia. The Fitness value of Australia was regarded as the average for Oceania, and the Fitness value of Brazil was regarded as the average for South America.

Since the number of genotypes from Africa did not exceed 300, the CoVFit model did not include data from African countries during training. Consequently, there are no Fitness prediction values for Africa. For the sake of completeness, the average Fitness prediction values of the 17 countries were used as the Fitness average for Africa. Therefore, when using CoVFit for model predictions, the S protein sequence data from Africa was input, and the average Fitness values of the 17 countries were used as the Fitness average for Africa.

Fitness values were also calculated for recent prevalent variants in North America. A total of 2,170 S protein amino acid sequences, with 543 variants, collected between April 1, 2024, and May 15, 2024 were utilized for the analysis.

### 2.5 Immune escape index

The CoVFit model can predict the relative binding affinities of different monoclonal antibodies (mAbs). By inputting a SARS-CoV-2 S protein sequence, the model can predict the relative binding affinities for 1,548 mAbs. We averaged these predicted affinity values and denoted the average as the IEI, which describes the immune escape capability of a variant. The higher the IEI, the greater the immune escape capability of the variant. IEI was calculated for the Global and North American dataset described in the prior section.

### 2.6 Constructing a null model for benchmark comparison of fitness and immune escape

To verify the statistical robustness of the Fitness and immune escape index (IEI) predictions for SARS-CoV-2 S protein sequences, we developed a null model that generates random sequences as a neutral evolution baseline. By comparing random data with real data, the null model aims to assess whether the observed results significantly deviate from neutral evolution expectations, thereby demonstrating the reliability of the computational outcomes. The null model is designed based on seven datasets, covering six continents (Africa, Asia, Europe, North America, Oceania, and South America) and an overall dataset (combining all six continents).

#### 2.6.1 Generation of random sequences under neutral evolution

**Figure 2:**
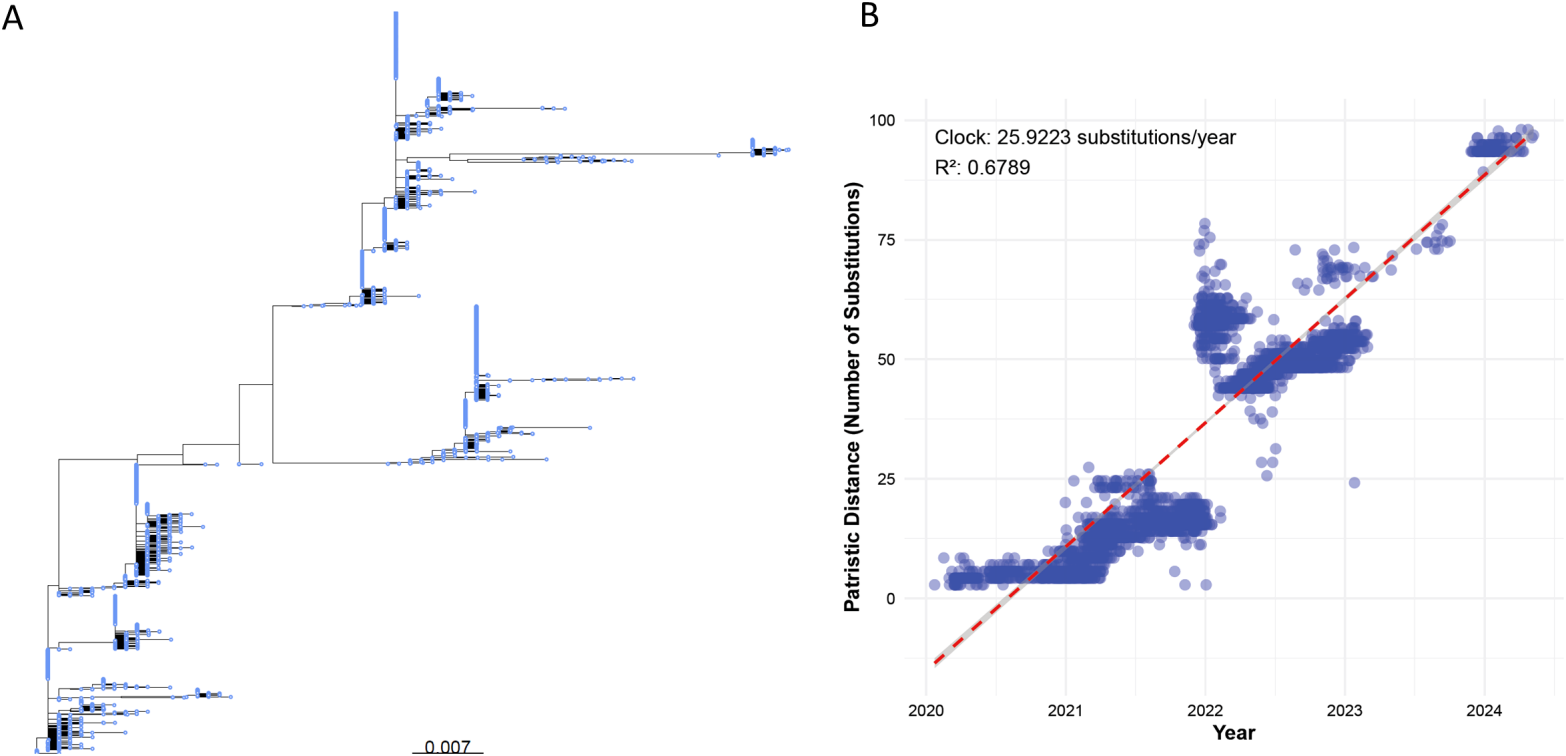
**(A)** Phylogenetic tree of a random subset of 5,000 global SARS-CoV-2 S protein sequences (sourced from NCBI SARS-CoV-2 Data Hub, 2020–2024), constructed using IQ-TREE v2.2.2.6 with the LG+I+G substitution model and the -czb flag to collapse short branches. The scale bar represents a genetic distance of 0.007 substitutions per site. **(B)** Scatter plot of patristic distances (calculated using the R package ape) versus sampling dates for the same 5,000 S protein sequences, aligned against the Wuhan-Hu-1 reference genome using MAFFT v7.520. Each point represents a sequence, with patristic distance measured as substitutions from the reference. The red dashed line shows the linear regression with a molecular clock rate of 25.9223 substitutions/year (R² = 0.6789).

The null model simulates the evolution of the S protein under neutral evolutionary conditions (unaffected by selection pressures from the host immune system, vaccination, or environmental factors), reflecting the accumulation of random mutations. This provides a statistical baseline to validate the Fitness/IEI of real sequences and the random mutatant sequence. Using the Wuhan-Hu-1 sequence (GenBank: MN908947.3, 1,273 amino acids) as a template, and based on the amino acid substitution rate from **Figure 2B** (25.9223 substitutions/year), neutral drift over 4.375 years (January 2020 to May 2024) is simulated through uniformly random mutation at each position.

#### 2.6.2 Calibration of evolutionary parameters

To reflect real evolutionary dynamics, key parameters are determined through the following steps:

Time Span: The simulation duration is set to 4.375 years, corresponding to the time window of this study;

Mutation Rate Calculation: Based on observed data, the S protein accumulates an average of 25.9223 amino acid mutations over 4.375 years, from which the per-site annual mutation rate is calculated:


$$\text{μ}=\frac{N_{mut}}{L}=\frac{25.9223}{1273}=0.02036\;\text{Mutation}/\text{site}/\text{year}$$

Where $N_{mut}$ is the total number of mutations per year, and L is the sequence length.

Through literature review, we found the proportion of neutral mutations in the S protein: approximately 10-30% overall, <10% in the RBD, and 20-40% in the non-RBD regions (2, 20, 27, 41, 42).

Neutral Proportion: Set 20% of mutations as driven by neutral evolution, adjusting the effective mutation rate to $\mu_{nertral}$ =μ×0.2;

Branch Length: The theoretical branch length is calculated as $\mu_{nertral}\times T$ , resulting in 0.0178.

#### 2.6.3 Evolutionary model configuration

**Substitution Model**: WAG amino acid substitution matrix (Whelan-And-Goldman model);

Phylogenetic Tree: Construct a parsimonious evolutionary tree;

Neutral Constraint: Set dN/dS = 1, retaining only neutral mutations.

#### 2.6.4 Variant generation

Perform *N* independent evolutionary simulations (e.g., 2,604 times, corresponding to a typical sampling scale in South America), with each simulation generating variants through the following steps:

- Use the Evolver module in Pyvolve (43) to evolve sequences along the specified phylogenetic tree;
- Randomly introduce mutations conforming to neutral constraints via a Poisson process;
- Record the final mutated sequences and save them in FASTA format.

The code for generating random mutation sequences can be found at:

https://github.com/pengsihua2023/SARS-CoV-2-Fitness-IEI/tree/main/code

#### 2.6.5 Kolmogorov-Smirnov test for assessing deviation of real variants from neutral evolution

We employed the CoVFit model to predict Fitness and Immune Escape Index (IEI) values for randomly generated variants, establishing an expected distribution under neutral evolution. Subsequently, we applied the Kolmogorov-Smirnov (KS) test (p < 0.05) to compare the distributions of real SARS-CoV-2 spike protein variants with those of the random variants, evaluating whether the observed evolutionary patterns significantly deviate from neutral expectations.

### 2.7 Outlier metadata verification and sensitivity analysis

To verify the metadata of two extreme outliers in North America (WZD59850.1 and WIJ15993.1), we examined their collection dates through the NCBI SARS-CoV-2 Data Hub. WZD59850.1 (reported date 2020-01-20, 66 mutations) and WIJ15993.1 (reported date 2020-02-02, 42 mutations) were inconsistent with the typical mutation counts of the same period (usually <10 (5)), suggesting potential date errors. To assess their impact, we removed these two samples (accounting for 0.0014% of the 140,000 variants in North America) and recalculated the molecular clock rate, as well as the mean Fitness and IEI values for North America.

### 2.8 Prediction study on the fitness and immune escape of the S protein in recent prevalent variants in North America

A total of 2,170 S protein amino acid sequences, with 543 variants, were downloaded from the United States between April 1, 2024, and May 15, 2024. We then conducted a prediction study on the fitness and immune escape capabilities of all the variants’ S proteins. It should be noted that the downloaded U.S. samples actually represent the sample size for all of North America.

### 2.9 Global retrospective analysis of S protein fitness and immune escape

We divided the time periods into three-month intervals and downloaded the S protein data for six continents from January 1, 2020, to May 15, 2024, removing duplicate sequences from the same time-period. We then conducted retrospective studies on the fitness and immune escape capabilities of the S proteins for each period and continent.

## 3 Results

### 3.1 Prediction study in America: January 1, 2024, to May 15, 2024

The CoVFit model was used to predict the Fitness and IEI values for the 543 variants in the United States, and the top 20 highest Fitness values were displayed in **Table 1**. The higher the Fitness value, the greater the transmission potential of the variant. The case with the highest Fitness value was from an Iowa case. The difference in Fitness values among these 20 variants was small, ranging from 0.921 to 0.925, and the differences in IEI were also minor, ranging from 0.581 to 0.589. Of these 20 variants, 11 lineages of SARS-CoV-2 are included. For example, lineages JN.1.16, JN.1.11.1, and JN.1.7 each had three variants included in the top 20 (**Table 1**). indicating that different numbers of mutations can lead to different Fitness values. This may be because different amino acid mutations may significantly alter the protein conformation, affecting the binding affinity of the S protein to the human ACE2 receptor.

**Table 1:** Top 20 predicted Fitness values and IEI for SARS-CoV-2 variants in the United States.

Variant Accession #	Lineage	Collection date	Fitness	IEI
XAN64366.1	JN.1.16	2024-04-29	0.925	0.585
XBA97060.1	JN.1.11.1	2024-05-09	0.924	0.584
XAW33708.1	JN.1.16	2024-05-06	0.924	0.584
XAU78949.1	JN.1.11.1	2024-04-20	0.924	0.584
XAU78842.1	JN.1.7	2024-04-17	0.923	0.584
XAJ36120.1	JN.1.11.1	2024-04-21	0.923	0.584
XAW33862.1	JN.1.4.2	2024-04-29	0.923	0.586
XAJ04662.1	JN.1.9	2024-04-10	0.923	0.585
XAN64414.1	JN.1	2024-04-25	0.922	0.589
XAW33674.1	BA.2.86.1	2024-05-04	0.922	0.589
XAO61989.1	JN.1	2024-04-02	0.922	0.581
XAJ04710.1	XDD	2024-04-14	0.922	0.588
XAU78878.1	JN.1.9	2024-04-24	0.921	0.588
WZH70794.1	JN.1.16	2024-04-08	0.921	0.581
XBA97012.1	JN.1.7.2	2024-04-30	0.921	0.588
XAW19132.1	JN.1.8.1	2024-04-17	0.921	0.588
XAJ29041.1	JN.1.7	2024-04-16	0.921	0.588
XAU78770.1	JN.1.7	2024-04-05	0.921	0.588
XAU78782.1	JN.1.4	2024-04-03	0.921	0.588
XAU78794.1	JN.1.4	2024-04-03	0.921	0.588

We then conducted a historical examination of the 11 lineages, identifying all global S protein variants from 2020 to 2024 (**Supplementary Table S2**). We found that the JN.1 lineage had the highest number of variants (n=1,082) while JN.1.7.2 had the fewest, with only 10 variants. In some lineages, there was an obvious difference between the Fitness and IEI of different variants. For example, the highest Fitness for JN.1.16 was 0.914, and the lowest was 0.863 (**Supplementary Table S2**).

In summary, we identified the top 20 variants as notable concerns in North America, with XAN64366.1 (JN.1.16 lineage) being the most significant due to its high Fitness (0.925) and IEI (0.585) values.

### 3.2 Retrospective study from January 1, 2020 to May 15, 2024

We analyzed the distribution of lineages and mutations within the variants, and identified the dominant lineage, mean mutations per variant, maximum and minimum of Fitness, and IEI for the variants, as well as its percentage among all lineages during each specific time period. Among the six continents, North America accounted for 95.46% of the cases and 89.66% of the variants. Therefore, we primarily present the analysis results for North America here (**Table 2**). The results of the other five continents are provided in the supplementary material (**Supplementary Tables S3–S7**).

**Table 2:** Profiles of SARS-Cov-2 variants in North America: lineage, mutation, fitness, and IEIs.

Time Period	Case/ Variant sequence	Dominant Lineage	Dominant Percentage	Unique Lineages	MMut	MaxFit	MFit	MaxIEI	MIEI
Jan-Mar, 2020	10,055/400	B.1	37.5%	73	2	0.963	0.227	0.591	0.171
Apr-Jun, 2020	19,662/968	B.1	35.85%	154	2	0.325	0.211	0.373	0.164
Jul-Sep, 2020	18,434/1,159	B.1	16.65%	169	2	0.329	0.212	0.513	0.164
Oct-Dec, 2020	45,478/3,264	B.1.2	30.91%	213	3	0.531	0.229	0.384	0.196
Jan-Mar, 2021	140,838/10,916	B.1.2	25.53%	283	6	0.534	0.286	0.466	0.258
Apr-Jun, 2021	177,356/11,233	B.1.1.7	39.6%	220	14	0.534	0.286	0.442	0.297
Jul-Sep, 2021	382,456/21,392	AY.44	11.53%	203	20	0.645	0.363	0.372	0.254
Oct-Dec, 2021	461,734/25,974	AY.103	17.9%	210	28	0.694	0.421	0.387	0.275
Jan-Mar, 2022	247,674/9,632	BA.1.1	39.11%	185	49	0.773	0.527	0.436	0.313
Apr-Jun, 2022	261,252/8,397	BA.2.12.1	31.19%	240	39	0.795	0.642	0.451	0.367
Jul-Sep, 2022	253,298/11,225	BA.5.2.1	12.71%	390	40	0.806	0.715	0.459	0.398
Oct-Dec, 2022	147,966/11,044	BQ.1.1	8.82%	626	42	0.837	0.755	0.476	0.424
Jan-Mar, 2023	90,796/8,895	XBB.1.5	23.66%	706	53	0.891	0.780	0.518	0.441
Apr-Jun, 2023	22,424/3,707	XBB.1.5	27.65%	562	50	0.901	0.808	0.532	0.459
Jul-Sep, 2023	38,369/5,797	FL.1.5.1	5.49%	629	64	0.911	0.857	0.532	0.489
Oct-Dec, 2023	44,399/6,486	HV.1	14.75%	534	63	0.924	0.895	0.551	0.522
Jan-Mar, 2024	26,275/3,216	JN.1	27.92%	247	72	0.913	0.901	0.545	0.536
Apr-May, 2024	2,170/543	JN.1	15.65	46	68	0.940	0.930	0.563	0.555

MMut, Mean Mutation; MaxFit, Maximum Fitness; MFit, Mean Fitness; MaxIEI, Maximum Immune Escape Index; MIEI, Mean Immune Escape Index.

#### 3.2.1 Unveiling the high evolutionary rate of the S protein

The phylogenetic tree (**Figure 2A**) illustrates distinct clusters and branching patterns, which are likely to correspond to different geographic regions or temporal clusters. This comprehensive phylogenetic analysis offers critical insights into the evolutionary dynamics of SARS-CoV-2 spike protein, enhancing our understanding of its proliferation and mutation over time.

**Figure 2B** shows that the molecular clock estimation reveals a substitution rate of approximately 25.9223 substitutions per year, indicating a high evolutionary rate for the S protein sequences sampled globally between 2020 and 2024. The scatter plot (**Figure 2B**) displays a general trend where patristic distances increase over time, fitting well with the estimated molecular clock rate (R² = 0.6789), suggesting a consistent rate of evolution across the observed period.

#### 3.2.2 Evolution of mutations in S protein variant sequences

**Figure 3:**
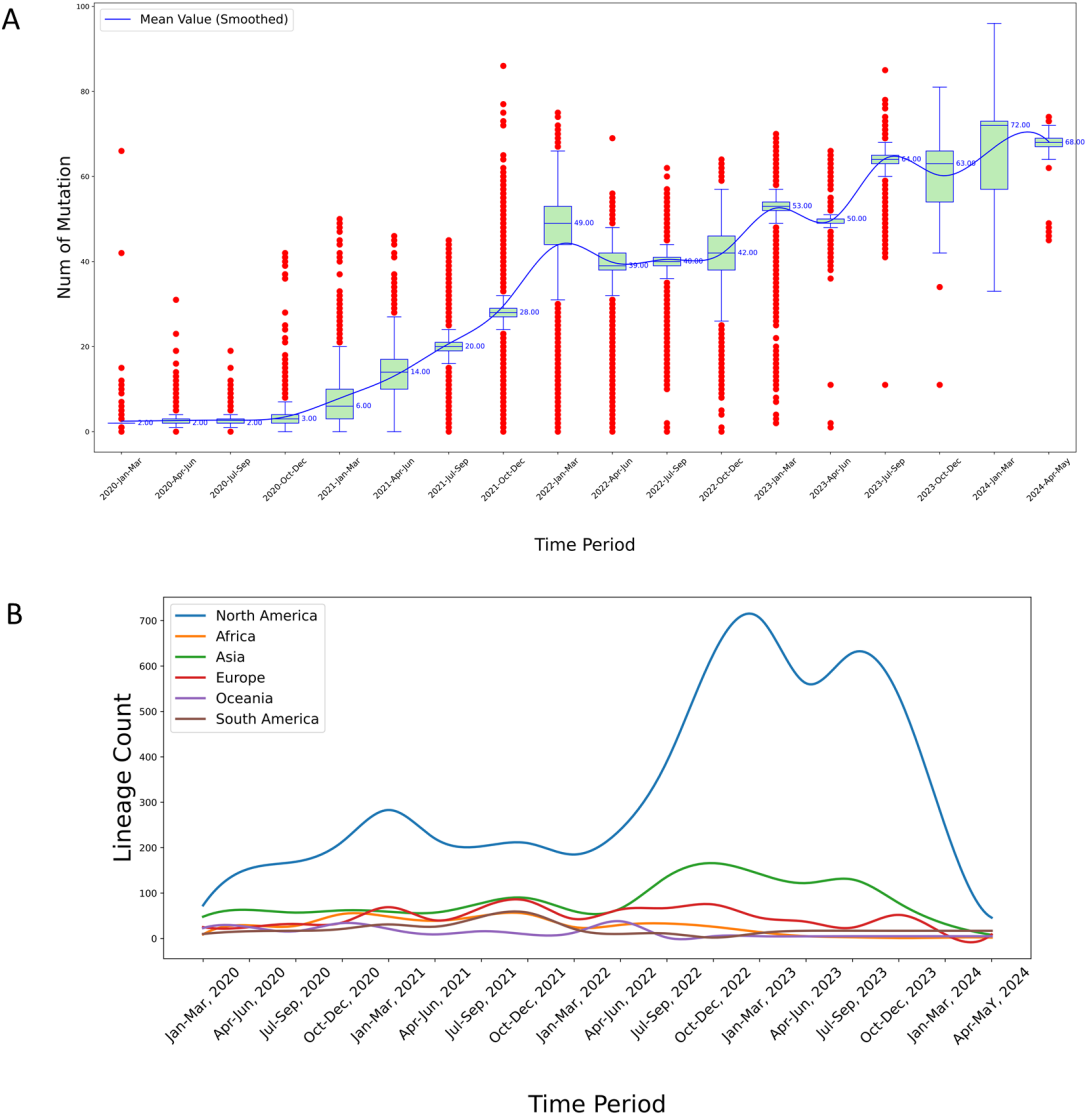
**(A)** Temporal analysis of mutational frequency per variant sequence in North America from 2020 to 2024. The red points in the plot represent outliers calculated based on quartiles. The median values are marked to the right of the green boxes, and the blue smoothed line represents the connected mean values across each time period, obtained through cubic spline interpolation; **(B)** Temporal trends in virus lineage counts across six continents from 2020 to 2024. This chart depicts the trends in the number of virus lineages observed on each of six continents over a five-year period, highlighting considerable fluctuations and a pronounced peak observed in the North America region. The variations and peak may suggest differences in viral evolution or the effectiveness of regional response strategies.

We found that the distribution of the number of mutations per variant during the entire SARS-CoV-2 pandemic could be divided into three distinct stages (**Figure 3A**, **Supplementary Figures S1–S5**). In North America, the first stage, spanning approximately from January 2020 to December 2021, marks a phase of rapid viral mutation. We observed a large number of outliers, representing sequences with extremely high numbers of mutations. During the middle stage of the pandemic, from January 2022 to March 2023, the total number of mutations continued to increase, however the number of outliers above the median decreased, indicating a slowdown in the rate of rapid mutations. This stage also recorded a substantial number of mutations below the median, suggesting that cases with fewer mutations from the earlier stage persisted. In the third stage, from April 2023 onward, the number of outliers, both those with fewer and those with a large number of mutations, significantly decreased, signaling a potential winding down of the pandemic (**Figure 3A**). Similar patterns were observed for the other five continents (**Supplementary Figures S1–S5**).

#### 3.2.3 Evolution of global SARS-CoV-2 lineage numbers over various time periods

Among the global dataset, 2,442 lineages were represented. The lineage counts in North America were consistently higher than those in other continents throughout the observed period (**Figure 3B**). This pronounced difference is likely attributable to the larger number of samples sequenced in North America, leading to the detection of more lineages.

Specifically, the lineage counts in North America exhibit three distinct peaks: The first peak occurred at the end of 2021, reaching a notable high; the second peak was observed at the end of 2022, again showing significant growth; and the third peak emerged around September 2023, indicating a third substantial increase.

In contrast, the lineage counts in other continents remained relatively stable over time. For instance: Europe and Asia showed relatively stable lineage counts throughout the period, with only minor fluctuations and no significant peaks comparable to those in North America; and Africa, Oceania, and South America exhibit relatively low lineage counts with minimal fluctuations, which might be due to fewer sequencing samples from these regions (**Figure 3B**).

Overall, the lineage counts in North America are significantly higher than those in other continents, with three notable peaks, reflecting differences in viral surveillance and sequencing sample sizes across regions.

#### 3.2.4 Comparison with Null model: Trends in fitness and immune escape

To contextualize the evolutionary trends of SARS-CoV-2 spike (S) protein sequences, we compared the Fitness and immune escape index (IEI) values of real sequences with those of random sequences generated based on a null model (see Section 2.6 for the methodology assumed for null model generation). The null model simulates neutral evolution by generating sequences with random mutations, providing a baseline to assess whether observed trends are driven by selective pressure or could arise randomly.

Globally, the distribution of Fitness values for real sequences (mean = 0.3849) differed significantly from that of random sequences (mean = 0.2046) (p < 0.001, KS test) (**Supplementary Table S8**). Similarly, the distribution of IEI values for real sequences (mean = 0.2894) showed a significant difference compared to random sequences (mean = 0.1895) (p < 0.001, KS test) (**Supplementary Table S9**). These results indicate that the Fitness and IEI values observed globally are substantially higher than expected under neutral evolution, suggesting that these traits may be driven by selective pressures, such as immune responses triggered by vaccination, natural infection, or environmental factors. Outlier analysis further supports this conclusion: real sequences exhibited extreme Fitness and IEI values (e.g., Fitness > 0.9, IEI > 0.6), which markedly exceed the typical range of random sequences. For instance, the highest Fitness value recorded in North America from April to May 2024 (0.940, **Table 2**) and the maximum IEI value (0.563) were far above the random sequence averages (0.2046 and 0.1895), reflecting selection for enhanced transmissibility and immune escape.

Across continents, trends in North America and Europe, where sequencing data are abundant, showed particularly pronounced differences between the Fitness and IEI distributions of real sequences and their corresponding random sequence distributions (p < 0.001, KS test). For example, North American Fitness increased from 0.227 in early 2020 to 0.930 in 2024 (**Table 2**), accompanied by a substantial sample size advantage (accounting for 95.46% of cases), indicating a strong evolutionary trend. Europe exhibited similarly significant differences, likely reflecting the impact of high vaccination rates and population density in accelerating variant evolution. In contrast, Africa and Oceania, with smaller sample sizes, showed smaller but still significant differences between real and random sequence distributions (p < 0.001, KS test), suggesting weaker yet still detectable selective pressures in these regions. South America and Asia displayed intermediate trends, correlated with regional public health measures and data coverage.

In summary, comparison with the null model demonstrates that the increases in Fitness and IEI of SARS-CoV-2 S protein sequences are the result of selective evolution rather than random effects from neutral mutations. The distribution of real sequences significantly deviates from the null model expectations, particularly in data-rich regions such as globally and North America, indicating that selective pressures (e.g., immune pressure and environmental factors) have shaped the virus’s evolutionary trajectory. This also underscores the statistical robustness of the fitness and immune escape predictions presented in this study.

#### 3.2.5 Temporal fitness evolution of SARS-CoV-2 S protein variants

**Figure 4:**
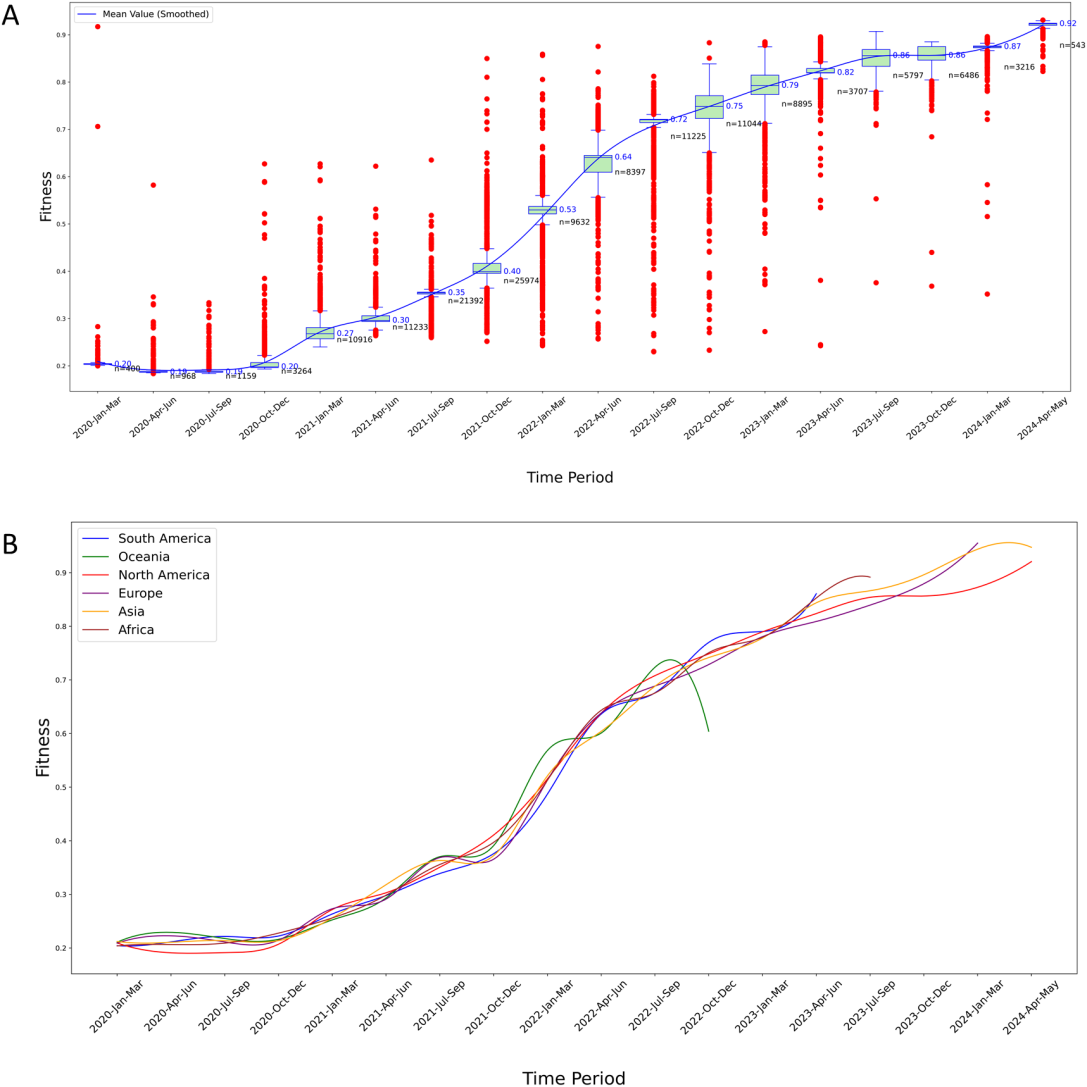
Temporal analysis of Fitness levels for S protein variants. **(A)** Fitness trends in North America: The red points in the plot represent outliers calculated based on quartiles. The median values are marked to the right of the green boxes, and the blue smoothed line represents the connected mean values across each time period, obtained through cubic spline interpolation. The n value marked in black in the figure indicates the sample size of variants in a certain time period; **(B)** Longitudinal comparison of fitness trends across six continents: This graph displays the smoothed mean Fitness values over time, highlighting significant regional variations and trends in the evolutionary adaptation of S protein variants. Each line represents a different continent, illustrating comparative rises in fitness levels, which may suggest differences in variant adaptability and potential immune escape efficiency.

Temporal Fitness over the four-and-a-half-year period from January 2020 to May 2024 can be roughly divided into three stages: The First stage (Jan 2020 - Mar 2022), the second stage (Mar 2022 - Mar 2023), and the third stage (Mar 2023 - May 2024) (**Figure 4A**).

**Figure 5:**
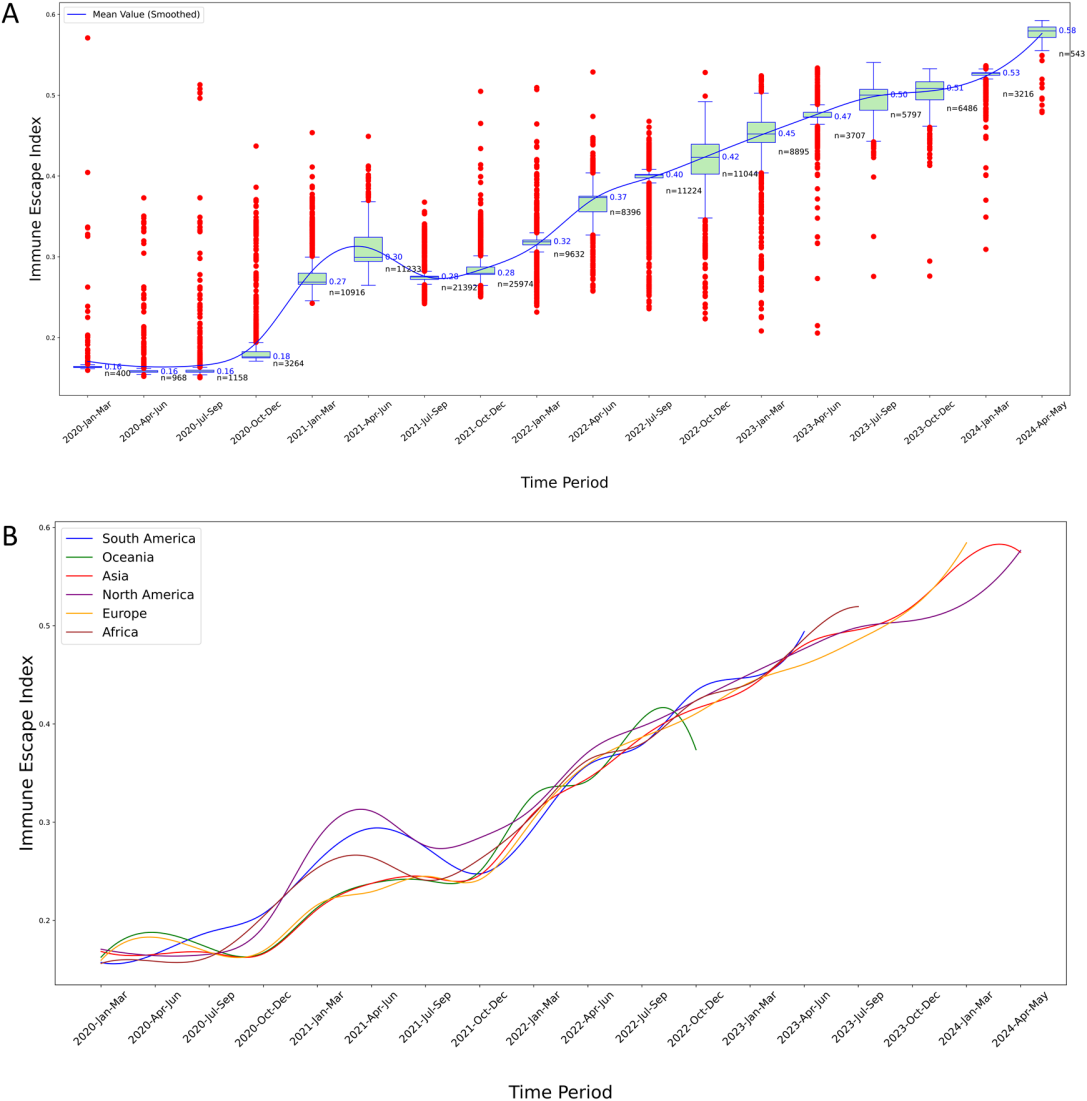
Temporal analysis of IEI for S protein variants. **(A)** IEI trends in North America: This boxplot graphically represents the distribution and temporal progression of the IEI for S protein variants in North America over a five-year period. The blue line traces the smoothed mean value of the IEI, illustrating an overall trend of increasing immune escape capabilities over time. The graph highlights the variability of the index, with some periods showing a high density of outlier values, suggesting episodes of significant evolutionary changes in the virus’s immune escape mechanisms. Each plotted point represents an individual variant’s measured IEI, providing a comprehensive overview of the changing landscape of viral resistance against immune responses over the specified timeframe; **(B)** Comparative analysis of IEI across six continents: This graph displays the smoothed mean values of the IEI for S protein variants across five continents, charting the trends from 2020 to 2024. Each colored line represents the trajectory of immune escape capabilities in South America, Oceania, North America, Europe, Asia, and Africa, indicating how these capabilities have evolved over the years. The chart highlights regional differences in the evolution of the virus’s immune escape mechanisms, with some continents showing more pronounced rises in immune escape indices than others. This visualization aids in understanding the geographical variation in viral adaptation and the potential implications for global public health strategies.

We found an increasing trend in the Fitness values of S protein variants over time. The virus with the lowest Fitness was from China (ancestral type, QZA85478.1, collect date 2020-02-23), with a fitness value of 0.234 (the lowest globally). Therefore, the Fitness values of all samples were compared to the wild type from China. In the early stage of the outbreak, the rate of change in Fitness (the slope of the curve) was not high, and the Fitness values were not high. In the middle stage, the rate of change in fitness sharply increased, which correlates with enhancement of the virus’s immune escape capability (**Figure 5A**). In the late stage, the growth rates of Fitness values and immune escape levels both slowed down, but their values had reached very high levels.

In the first stage, there were many outliers with Fitness values higher than the maximum value of the boxplot, which is a significant characteristic of the early outbreak of SARS-CoV-2. The numerous outliers indicated that the virus evolved rapidly during the early stage of the outbreak, with a large accumulation of mutations. The more mutations there were, the higher the Fitness of the S protein, resulting in many variants with Fitness values significantly exceeding the mean Fitness during this phase (**Figure 4A**).

In the second stage, there was a notable decrease in the number of outliers exceeding the boxplot’s maximum value, while a substantial proportion of outliers fell below the minimum value. Throughout this stage, not only did these low-Fitness variants continue to circulate, but high-Fitness variants also emerged more frequently. The Fitness levels increased at the fastest rate during this phase, as evidenced by the steep slope of the smoothed mean Fitness curve (**Figure 4A**).

In the third stage, the prevalence of low-Fitness variants was very rare, and there were also fewer variants with Fitness values above the average, suggesting that this is a major characteristic of the end of the pandemic. During this stage, although the Fitness values were high, the slope of the smoothed Fitness curve decreased significantly. At the same time, there were very few variants with Fitness values above the mean, indicating that the rate of virus evolution had slowed down (**Figure 4A**).

Similar characteristics were also observed in the results of the other five continents (**Figure 4B**, **Supplementary Figures S6–S10**).

Overall, the above results highlight the evolutionary trends in the Fitness of S protein variants in all the six continents, demonstrating an increase in Fitness over time.

#### 3.2.6 Temporal evolution of immune escape capacity in SARS-CoV-2 S protein variants

The smoothed mean IEI showed a general increasing trend over time, indicating that the immune escape capability of S protein variants has progressively improved (**Figure 5A**). Early time periods exhibited lower IEI values with significant variability and numerous outliers, suggesting diverse immune escape capabilities among early variants. As time progressed, the IEI values increased, with the variability and number of outliers decreasing, reflecting more consistent and higher immune escape capabilities among newer variants.

In the IEI curves, the trajectories for all six continents generally exhibited an upward trend, yet each showed one or more periods of decline or stagnation over shorter intervals. For instance, the IEI for North America reached a local trough in 2021 (**Figure 5A**).

**Figure 5B** highlights the evolutionary trends in the immune escape capabilities of S protein variants across the six continents, showing an increase in IEI over time with reduced variability among more recent variants. However, in 2021, the IEI for the other five continents also exhibited short-term declines, similar to the results observed in North America (**Figure 5B**, **Supplementary Figures S11–S15**).

#### 3.2.7 Sensitivity analysis of outlier impact

For the two outliers in North America (WZD59850.1 and WIJ15993.1, see **Figures 4A**, **5A**), metadata verification indicated that their collection dates in early 2020 were inconsistent with their high mutation counts (66 and 42), possibly due to recording errors. After their removal, the molecular clock rate remained at 25.9223 substitutions per year (change <0.0001%), and the mean Fitness and IEI values in North America showed no significant changes (both <0.001%). Thus, the impact of these two outliers was significantly diluted and did not alter the main conclusions.

## 4 Discussion

This retrospective study elucidates the dynamic evolution of the SARS-CoV-2 spike protein from January 2020 to May 2024, revealing significant shifts in viral adaptability and immune escape capabilities. Utilizing advanced protein language models, our research emphasizes the role of genetic mutations in shaping the trajectory of the COVID-19 pandemic, revealing intricate mechanisms by which the virus adapts to the human immune system.

In this study, we constructed a null model based on data from six continents and the overall dataset, providing a baseline for evaluating the fitness and immune escape trends of SARS-CoV-2 S protein variants. Significant deviations of the adaptability and immune escape index (IEI) of real sequences from the expectations of the null model (see Section 3.3) suggest that adaptive evolution plays a critical role in enhancing viral transmissibility and immune escape capabilities. We incorporated an amino acid substitution rate (25.9223 substitutions per year) into the null model design and employed a uniform random mutation generation approach, further confirming that the observed evolutionary trends are not merely a simple product of neutral drift. The sequence variation model proposed by Lee et al., based on the frequency distribution of actual variants, could be integrated in the future to optimize the null model, better capturing mutation hotspots and variant competition effects, thus deepening the understanding of viral evolution mechanisms (44).

Geographic and temporal variations indicate that the virus’s adaptability and immune escape indices vary with environmental conditions and the genetic diversity of host populations. These variations might reflect different evolutionary pressures, such as those exerted by high population densities accelerating virus mutation and transmission, while extensive public health interventions could limit the spread of these variants (45, 46).

Compared to the original samples discovered in Wuhan, the top 20 SARS-CoV-2 variants in North America have shown higher adaptability and immune evasion capabilities, suggesting that the virus has reached a high level of adaptation in human hosts. However, the pace of the virus’s evolution has slowed, likely because it has found a relatively stable state of adaptation within the current biological and social environments. Nevertheless, whether it will gradually evolve into a seasonal virus, much like seasonal influenza requires ongoing observation of the virus’s long-term behavioral patterns and its impact on public health. Thus, claiming that the virus will evolve into a seasonal flu remains premature and requires further scientific evidence to support such a prediction.

We observed that the IEI for North America reached a local trough in 2021. This could be attributed to several factors. Firstly, starting in 2021, various public health interventions were implemented by countries and health organizations, such as lockdowns and travel restrictions (47, 48). These measures likely curtailed the spread of the virus, particularly variants with high immune escape capabilities (49) to accumulate new mutations to evade immune. Secondly, with the widespread rollout of vaccines, variants with higher immune escape capabilities may have been effectively suppressed for a period of time, resulting in a temporary decrease in the overall IEI (50). However, despite the significant increase in global vaccination rates over time, the IEI of the virus has not shown a sustained downward or stagnant trend. This indicates that the continuous accumulation of mutations in the S protein has enhanced the virus’s adaptability, leading to a persistent rise in immune escape capabilities. The rapid evolution of the virus means that newly developed vaccines quickly become less effective, thereby contributing to the ongoing increase in the IEI (21).

Additionally, our findings during the study period (from Apr 1, 2024 to May 15, 2024) indicated a higher adaptability of spike protein variants in North America as of early 2024, suggesting that the virus in this region may be evolving towards a more stable phase of adaptability. This stabilization might signal the virus transitioning towards an endemic phase, potentially manifesting a periodic outbreak pattern similar to seasonal influenza (46, 51, 52).

Although this study is primarily based on North American data, we believe its conclusions have global applicability. The high sequencing coverage in North America makes it a robust foundation for observing the evolutionary dynamics of SARS-CoV-2, such as the increased Fitness of the JN.1 lineage. The World Health Organization (WHO, 2024) reported that, as of April 2024, more than 94% of global SARS-CoV-2 sequences were derived from JN.1 (see https://www.who.int/news/item/26-04-2024-statement-on-the-antigen-composition-of-covid-19-vaccines), and this trend was further confirmed in December 2024, when all circulating variants were descendants of JN.1 (see https://www.who.int/news/item/23-12-2024-statement-on-the-antigen-composition-of-covid-19-vaccines). This global consistency, alongside earlier observations of mutations like D614G and N439K spreading across multiple regions (2), indicates that the evolutionary trends observed in North America have been validated in other regions. Furthermore, the universality of immune selection pressure, such as the immune escape properties of JN.1, aligns with findings that immune-driven evolution is a global phenomenon (2), further supporting the global relevance of our conclusions (see https://www.who.int/news/item/23-12-2024-statement-on-the-antigen-composition-of-covid-19-vaccines). Notably, despite increasing global vaccination coverage, the virus’s IEI continues to rise (49). This highlights the high adaptability of SARS-CoV-2 under immune pressure and its capacity to accumulate new mutations to evade immune responses (45). Therefore, ongoing genetic surveillance and timely adjustments in vaccine strategies are crucial to manage potential outbreaks.

This study employed a protein language model to conduct a retrospective analysis of the spike protein of the SARS-CoV-2 virus. Methodologically, CoVFit utilized historical data to develop a deep learning model, on which basis our study predicted the protein fitness and immune evasion capabilities of historical spike protein (S protein) sequences. To address potential inquiries, we clarify that the training data for the CoVFit model comprised 21,751 genotype-fitness data points, covering 12,914 genotypes across 17 countries (19). Due to the presence of different mutations or variant combinations that can constitute distinct genotypes, many of the genotypes contain repeated mutations. Thus, the number of variants used in training the CoVFit model is estimated to be in the hundreds to thousands, which can be precisely quantified using CoVFit’s original dataset. Additionally, in our study, the total number of global variants analyzed was 160,892, and the variant amino acid sequences used did not include any uncertain ‘X’ entries. Consequently, in this retrospective analysis, only about 2% of the data overlaps with the model training data. To ensure the integrity of the sample, we did not exclude this very small proportion of overlapping data. Therefore, although the retrospective study may include a minimal portion of the data used during model training, this does not affect the primary conclusions drawn from our research.

There are two data points from North America that are exceptionally high in terms of the number of mutations, Fitness values, and IEI values. The Fitness/IEI/Mutation values are 0.944/0.571/66 (WZD59850.1, JN.1) and 0.712/0.404/42 (WIJ15993.1, BA.4.6), with collection dates of 2020-01–20 and 2020-02–02 respectively. Firstly, we speculate that the collection dates of these two samples may have been recorded incorrectly. This is because other samples with a Fitness value greater than 0.9 occurred after April 2023, and the remaining samples with Fitness values above 0.7 appeared after October 2021.

In the case of WZD59850.1, if the recorded collection date is correct, this would imply that the sample underwent an astounding number of 66 mutations in an incredibly short period during the early stages of the virus outbreak. The source of this sample may not be Wuhan, China, and it could represent mutations accumulated locally in the USA over 2–3 years, though further phylogenetic analysis is needed to confirm this hypothesis.

## 5 Limitations of this study

Despite providing comprehensive analysis, this study has several limitations that need to be considered.

First, the accuracy of our predictions largely depends on the quality of the data used, and our dataset, comprising 2.5 million sequences (approximately 160,892 variants), may still exhibit biases due to North America’s overrepresentation. This could potentially skew the understanding of global viral evolution patterns. For instance, although we addressed two North American outliers (WZD59850.1 and WIJ15993.1, see Sections 2.7 and 3.2.7) through sensitivity analysis, confirming their negligible impact on the results (molecular clock rate variation <0.001%, Fitness and IEI mean variation <0.01%), the extremely limited sample sizes from non-North American regions (e.g., Africa, Oceania, and South America), such as fewer than 300 variants in Africa (see Section 2.4), may result in insufficient detection of region-specific evolutionary trends.

Second, although the CoVFit protein language model represents significant progress in predicting fitness and immune escape capabilities, it is fundamentally limited by the quality and diversity of its training data. The model was fine-tuned on 21,751 genotype-fitness data points from 17 countries (Section 2.4), but changes in viral properties not adequately captured in these training sequences—such as complex mutation interactions (epistasis) or rare variant effects—might not be accurately reflected in the model’s predictions. Additionally, while CoVFit was pre-trained on 1,506 types of coronaviruses, its ability to fully capture SARS-CoV-2-specific evolutionary pressures may still be constrained by the representativeness of the training dataset.

Lastly, while we strive for precision in our analyses, computational predictions of immune escape capabilities cannot fully substitute for empirical validation in a laboratory setting. Continuous verification of computational results with experimental data is necessary to ensure the accuracy and relevance of the predictions made.

These limitations underscore the importance of ongoing research, continuous data collection, and model updates. In the future, the predictive accuracy and utility of computational tools in virology can be enhanced by increasing sequence data from non-North American regions, improving the model’s ability to capture mutation interactions, and integrating *in vitro* experimental validation to complement computational predictions. Specifically, future studies could employ pseudovirus neutralization assays to experimentally validate the predicted fitness and immune escape capabilities of SARS-CoV-2 variants, providing a more robust assessment of the model’s predictions.

## 6 Conclusions

Our retrospective analysis of the SARS-CoV-2 spike protein from January 2020 to May 2024, using the CoVFit protein language model, has elucidated the virus’s remarkable evolutionary dynamics. The study reveals a significant increase in Fitness (from a mean of 0.227 in 2020 to 0.930 in 2024) and immune escape index (IEI, from 0.171 to 0.555) for North America samples.

Globally, the comparison of Fitness and IEI between real mutants and random mutants, which were generated by the null model, demonstrated statistical significance (real mutant Fitness 0.3849 vs. random mutant 0.2046, p < 0.001, KS test; real mutant IEI 0.2894 vs. random mutant 0.1895, p < 0.001, KS test), indicating strong selective pressure.

Globally, the JN.1 lineage dominated (94% of sequences by April 2024), underscoring its evolutionary advantage.

CoVFit provides critical insights into SARS-CoV-2 evolution, offering a robust tool for understanding how genetic variations enhance viral fitness and immune resistance. These findings underscore the virus’s ability to adapt despite widespread interventions, emphasizing the urgent need for continuous genetic surveillance and adaptive public health strategies. The observed stabilization in adaptability, particularly in North America, suggests a potential transition toward endemicity, necessitating region-specific responses to optimize control efforts. Advanced computational tools like CoVFit are indispensable for predicting evolutionary trends, supporting vaccine development, and enhancing global pandemic preparedness.

## Data Availability

Publicly available datasets were analyzed in this study. This data can be found here: https://github.com/pengsihua2023/VFIEI-SARS-cov-2.
