# Supplementary material for: Evolving fitness and immune escape: a retrospective analysis of SARS-CoV-2 spike protein (2020-2024) using protein language model
Source: Front Immunol. 2025 Jun 18;16:1576414. doi: 10.3389/fimmu.2025.1576414 (PMC12213458; doi:10.3389/fimmu.2025.1576414)
Supplement: Supplementary file 1 [file DataSheet1.pdf]

Supplementary Information

Table S1 Dataset of SARS-CoV-2 S protein sequences downloaded

Time period	Africa (case/variant)	Asia (case/variant)	Europe (case/variant)	North America (case/variant)	Oceania (case/variant)	South America (case/variant)
Jan-Mar, 2020	94/13	2,274/214	1,406/104	10,055/400	854/64	149/15
Apr-Jun, 2020	778/105	2,808/347	1,444/126	19,662/968	977/68	410/44
Jul-Sep, 2020	645/100	2,100/242	1,675/145	18,434/1,159	8,240/229	116/32
Oct-Dec, 2020	1,192/268	2,661/348	1,885/224	45,478/3,264	425/63	319/95
Jan-Mar, 2021	1,589/393	1,248/323	3,319/451	140,838/10,916	98/36	356/138
Apr-Jun, 2021	1,447/358	2,157/469	1,584/359	177,356/11,233	61/23	2,273/489
Jul-Sep, 2021	1,933/491	4,703/812	3,416/546	382,456/21,392	2,017/47	4,771/824
Oct-Dec, 2021	1,281/235	3,969/730	3,069/617	461,734/25,974	2,551/92	4,147/760
Jan-Mar, 2022	720/95	8,648/727	1,939/271	247,674/9,632	2,611/119	1,948/140
Apr-Jun, 2022	854/157	2,777/367	2,168/300	261,252/8,397	2,603/217	88/30
Jul-Sep, 2022	298/75	4,178/605	1,162/214	253,298/11,225	2/2	106/32
Oct-Dec, 2022	250/61	2,268/534	379/144	147,966/11,044	18/6	17/5
Jan-Mar, 2023	35/21	1,087/324	121/64	90,796/8,895	/	49/24
Apr-Jun, 2023	11/7	908/301	146/74	22,424/3,707	/	76/38
Jul-Sep, 2023	4/3	667/317	44/31	38,369/5,797	/	/
Oct-Dec, 2023	/	313/156	180/101	44,399/6,486	/	/
Jan-Mar, 2024	/	160/79	43/17	26,275/3,216	/	/
Apr-May, 2024	/	10/9	/	2,170/543	/	/
Subtotal	11,131/2,382	43,249/6,904	23,980/3,788	2,390,636/144,248	20,457/966	14,825/2,604
Total	2,504,278/160,892*, including 135,492 unique variant sequences.					

* The variant number is the result of accumulation over time periods, so the real variants are smaller than this number.

Table S2 Historical examination of fitness and IEI across 11 lineages of SARS-CoV-2 variants

	Variants	Max Fitness/Date/Country Accession	Min Fitness/Date/Country Accession	Max IEI/Date/Country Accession	Min IEI/Date/Country Accession
JN.1.16	88	0.914|2024-03-19|USA WZC46762.1	0.863|2024-05-02|USA XAX97799.1	0.603|2024-03-19|USA WZC46762.1	0.555|2024-05-02|USA XAX97799.1
JN.1.11.1	33	0.908|2024-05-01|USA XBA92584.1	0.885|2024-05-11|USA XBG56702.1	0.655|2024-04-22|USA XAN95867.1	0.630|2024-05-11|USA XBG56702.1
JN.1.7	143	0.913|2024-04-15|USA XAN71841.1	0.827|2024-04-01|USA WZH69489.1	0.616|2024-05-15|USA XBL92471.1	0.553|2024-04-01|USA WZH69489.1
JN.1.4.2	99	0.915|2024-05-15|USA XBL92342.1	0.913|2023-12-07|USA WQQ58013.1	0.615|2024-02-15|USA WWQ17088.1	0.563|2024-01-16|USA WWZ24569.1
JN.1.9	63	0.907|2024-04-10|USA XAJ04662.1	0.871|2024-02-14|USA WWQ16291.1	0.599|2024-01-17|USA WVH24328.1 |	0.569|2024-02-14|USA WWQ16291.1
JN.1	1082	0.890|2023-12-09|USA WVH05687.1	0.811|2024-01-28|USA WVW94984.1	0.623|2023-12-23|USA WWQ99983.1	0.506|2024-03-29|USA WYX99223.1
BA.2.86.1	69	0.903|2024-01-02| USA WWZ24049.1	0.860|2024-02-03| USA WWB25296.1	0.624/2024-01-22|Japan BFH89706.1	0.582|2024-02-03|USA WWB25296.1
XDD	16	0.903|2024-04-14|USA XAJ04710.1	0.899|2024-01-19|USA WZC33827.1	0.601|2024-02-15|USA WXH69581.1	0.588|2023-12-22|USA WVQ25143.1
JN.1.7.2	10	0.904|2024-04-30|USA XBA04631.1	0.899|2024-03-19|USA WYX95543.1	0.594|2024-04-04|USA XAO62227.1	0.580|2024-03-19|USA WYX95543.1
JN.1.8.1	121	0.902|2024-04-22|USA XAN95821.1	0.800|2024-03|USA WYK03097.1	0.680|2024-02-12|USA WWZ27898.1	0.585|2023-12-26|USA WRO52942.1
JN.1.4	562	0.914|2023-12-14|USA WRI41262.1	0.748|2024-01-03|USA WXB52480.1	0.701|2024-01-05|USA WWA06107.1	0.598|2024-01-23|USA WVO05100.1

Table S3 Profiles of SARS-cov-2 variants in Africa: lineage, mutation details, Fitness, and IEIs

	Case / Variant sequence	Dominant Lineage	Dominant Percentage*	Unique Lineages	MMut	MaxFit	MFit	MaxIEI	MIEI
Jan-Mar, 2020	94/13	B.1	30.77%	9	2	0.213	0.204	0.168	0.156
Apr-Jun, 2020	778/105	B.1	32.38%	29	2	0.306	0.207	0.299	0.158
Jul-Sep, 2020	645/100	B.1	34.0%	28	2	0.283	0.210	0.349	0.163
Oct-Dec, 2020	1,192/268	B.1	23.51%	54	9	0.370	0.228	0.350	0.204
Jan-Mar, 2021	1,580/393	B.1.1.7	20.36%	48	9	0.372	0.257	0.369	0.253
Apr-Jun, 2021	1,447/358	B.1.1.7	22.91%	39	11	0.376	0.299	0.371	0.264
Jul-Sep, 2021	1,933/491	AY.36	23.63%	50	11	0.388	0.356	0.350	0.241
Oct-Dec, 2021	1,281/235	B.1.617.2	14.89%	54	12	0.738	0.397	0.415	0.262
Jan-Mar, 2022	720/95	BA.1.1	37.89%	25	38	0.771	0.513	0.437	0.308
Apr-Jun, 2022	854/157	BA.2	24.20%	30	33	0.737	0.643	0.415	0.363
Jul-Sep, 2022	298/75	BA.5.2.1	16.00%	33	35	0.831	0.676	0.472	0.380
Oct-Dec, 2022	250/61	BQ.1.1	22.95%	26	38	0.813	0.751	0.459	0.424
Jan-Mar, 2023	35/21	BQ.1.1	14.29%	14	38	0.842	0.780	0.478	0.441
Apr-Jun, 2023	11/7	XBB.1.16.2	28.57%	5	45	0.886	0.853	0.508	0.487
Jul-Sep, 2023	4/3	XBB.1.16.2	33.33%	3	45	0.938	0.892	0.564	0.519
Oct-Dec, 2023	/	/	/	/	/	/	/	/	/
Jan-Mar, 2024	/	/	/	/	/	/	/	/	/
Apr-May, 2024	/	/	/	/	/	/	/	/	/

* Dominant Percentage: (num of dominant lineage) / (num of variant);

Mean Mutations per variant sequence: MMut; Maximum Fitness: MaxFit; Mean Fitness: MFit;

Maximum Immune Escape Index: MaxIEI; Mean Immune Escape Index: MIEI

Table S4 Profiles of SARS-cov-2 variants in Asia: lineage, mutation details, Fitness, and IEIs

	Case / Variant sequence	Dominant Lineage	Dominant Percentage*	Unique Lineages	MMut	MaxFit	MFit	MaxIEI	MIEI
Jan-Mar, 2020	2,274/214	B.1.1.63	14.41%	48	2	0.373	0.212	0.489	0.168
Apr-Jun, 2020	2,808/347	B.1	13.21%	63	2	0.328	0.211	0.368	0.165
Jul-Sep, 2020	2,100/242	B.1.1.63	13.88%	57	2	0.370	0.214	0.330	0.167
Oct-Dec, 2020	2,661/348	B.1.1.312	9.48%	62	3	0.373	0.214	0.348	0.166
Jan-Mar, 2021	1,248/323	B.1.1.7	16.10%	59	5	0.595	0.256	0.402	0.211
Apr-Jun, 2021	2,157/469	B.1.617.2	31.56%	57	11	0.700	0.318	0.389	0.238
Jul-Sep, 2021	4,703/812	B.1.617.2	29.56%	78	15	0.612	0.363	0.358	0.244
Oct-Dec, 2021	3,969/730	B.1.617.2	20.82%	89	12	0.600	0.372	0.350	0.246
Jan-Mar, 2022	8,648/727	BA.2	23.38%	60	32	0.760	0.521	0.431	0.309
Apr-Jun, 2022	2,777/367	BA.2	28.07%	66	32	0.707	0.604	0.396	0.345
Jul-Sep, 2022	4,178/605	BA.5.2	23.14%	136	37	0.773	0.688	0.438	0.386
Oct-Dec, 2022	2,268/534	XBB.1	11.99%	166	37	0.840	0.742	0.474	0.416
Jan-Mar, 2023	1,087/324	BF.5	7.41%	142	41	0.876	0.778	0.504	0.438
Apr-Jun, 2023	908/301	XBB.1.16	8.31%	122	43	0.898	0.844	0.515	0.480
Jul-Sep, 2023	667/317	XBB.1.16	6.94%	130	44	0.935	0.866	0.547	0.496
Oct-Dec, 2023	313/156	HK.3	7.69%	78	56	0.928	0.896	0.551	0.520
Jan-Mar, 2024	160/79	JN.1	20.25%	32	66	0.963	0.944	0.592	0.569
Apr-May, 2024	10/9	XDQ.1	30.00%	8	66	0.958	0.947	0.590	0.574

* Dominant Percentage: (num of dominant lineage)/(num of variant);

Mean Mutations per variant sequence: MMut; Maximum Fitness: MaxFit; Mean Fitness: MFit;

Maximum Immune Escape Index: MaxIEI; Mean Immune Escape Index: MIEI

Table S5 Profiles of SARS-cov-2 variants in Europe: lineage, mutation details, Fitness, and IEIs

	Case / Variant sequence	Dominant Lineage	Dominant Percentage*	Unique Lineages	MMut	MaxFit	MFit	MaxIEI	MIEI
Jan-Mar, 2020	1,406/104	B.1	47.12%	25	2	0.270	0.209	0.255	0.159
Apr-Jun, 2020	1,444/126	B.1	55.56%	25	2	0.380	0.223	0.309	0.183
Jul-Sep, 2020	1,675/145	B.1.160	37.24%	32	3	0.311	0.211	0.339	0.168
Oct-Dec, 2020	1,885/224	B.1.160	38.39%	35	3	0.328	0.214	0.352	0.169
Jan-Mar, 2021	3,319/451	B.1.1.7	29.05%	69	5	0.416	0.273	0.343	0.216
Apr-Jun, 2021	1,584/359	B.1.1.7	71.87%	40	11	0.378	0.291	0.370	0.229
Jul-Sep, 2021	3,416/546	AY.43	28.02%	68	11	0.507	0.369	0.336	0.245
Oct-Dec, 2021	3,069/617	AY.43	22.20%	83	12	0.597	0.366	0.370	0.241
Jan-Mar, 2022	1,939/271	BA.2	25.46%	43	33	0.627	0.513	0.359	0.304
Apr-Jun, 2022	2,168/300	BA.2	44.33%	64	34	0.751	0.638	0.421	0.359
Jul-Sep, 2022	1,162/214	BA.5.1	21.03%	67	35	0.804	0.689	0.457	0.386
Oct-Dec, 2022	379/144	BQ.1.1	11.81%	75	36	0.838	0.729	0.475	0.411
Jan-Mar, 2023	121/64	BQ.1.1	10.77%	46	40	0.955	0.781	0.576	0.442
Apr-Jun, 2023	146/74	XBB.1.9.1	22.97%	37	41	0.884	0.809	0.509	0.461
Jul-Sep, 2023	44/31	XBB.1.9.1	11.43%	24	42	0.931	0.840	0.559	0.486
Oct-Dec, 2023	180/101	XBB.2.3.11	8.82%	52	44	0.955	0.879	0.588	0.519
Jan-Mar, 2024	43/17	JN.1	38.89%	9	66	0.962	0.955	0.594	0.584
Apr-May, 2024	/	/	/	/	/				

* Dominant Percentage: (num of dominant lineage)/(num of variant);

Mean Mutations per variant sequence: MMut; Maximum Fitness: MaxFit; Mean Fitness: MFit;

Maximum Immune Escape Index: MaxIEI; Mean Immune Escape Index: MIEI

Table S6 Profiles of SARS-cov-2 variants in Oceania: lineage, mutation details, Fitness, and IEIs

	Case / Variant sequence	Dominant Lineage	Dominant Percentage*	Unique Lineages	MMut	MaxFit	MFit	MaxIEI	MIEI
Jan-Mar, 2020	854/64	D.2	31.25%	23	2	0.369	0.211	0.250	0.163
Apr-Jun, 2020	977/68	D.2	41.18%	25	2	0.383	0.229	0.244	0.188
Jul-Sep, 2020	8,240/229	D.2	92.58%	16	3	0.369	0.218	0.326	0.172
Oct-Dec, 2020	425/63	D.2	33.33%	34	3	0.369	0.216	0.337	0.167
Jan-Mar, 2021	98/36	B.1.1.7	30.56%	21	7.5	0.369	0.253	0.348	0.213
Apr-Jun, 2021	61/23	B.1.1.7	52.17%	9	11	0.373	0.293	0.367	0.238
Jul-Sep, 2021	2,017/47	AY.39.1.1	59.57%	16	12	0.379	0.370	0.248	0.241
Oct-Dec, 2021	2,551/92	AY.39.1.1	88.04%	9	12	0.595	0.391	0.338	0.250
Jan-Mar, 2022	2,611/119	BA.2.10	32.77%	13	33	0.672	0.568	0.382	0.328
Apr-Jun, 2022	2,603/217	BA.2	51.61%	38	32	0.702	0.601	0.391	0.342
Jul-Sep, 2022	2/2	BA.2.75.2	50.00%	2	37	0.748	0.724	0.420	0.405
Oct-Dec, 2022	18/6	B.1.1.136	33.33%	5	38	0.801	0.604	0.455	0.374
Jan-Mar, 2023	/	/	/	/	/	/	/	/	/
Apr-Jun, 2023	/	/	/	/	/	/	/	/	/
Jul-Sep, 2023	/	/	/	/	/	/	/	/	/
Oct-Dec, 2023	/	/	/	/	/	/	/	/	/
Jan-Mar, 2024	/	/	/	/	/	/	/	/	/
Apr-May, 2024	/	/	/	/	/	/	/	/	/

* Dominant Percentage: (num of dominant lineage)/(num of variant);

Mean Mutations per variant sequence: MMut; Maximum Fitness: MaxFit; Mean Fitness: MFit;

Maximum Immune Escape Index: MaxIEI; Mean Immune Escape Index: MIEI

Table S7 Profiles of SARS-cov-2 variants in South America: lineage, mutation details, Fitness, and IEIs

	Case / Variant sequence	Dominant Lineage	Dominant Percentage*	Unique Lineages	MMut	MaxFit	MFit	MaxIEI	MIEI
Jan-Mar, 2020	149/15	B.1	26.67%	10	2	0.235	0.204	0.195	0.157
Apr-Jun, 2020	410/44	B.1	22.73%	16	2	0.324	0.211	0.300	0.166
Jul-Sep, 2020	116/32	B.1.1.28	12.50%	17	2	0.362	0.222	0.348	0.188
Oct-Dec, 2020	319/95	B.1.1.28	17.89%	21	3	0.357	0.222	0.349	0.207
Jan-Mar, 2021	356/138	P.2	20.29%	31	4	0.329	0.263	0.344	0.260
Apr-Jun, 2021	2,273/489	P.1	64.49%	26	13	0.375	0.298	0.345	0.294
Jul-Sep, 2021	4,771/824	P.1	33.13%	48	12	0.387	0.339	0.337	0.275
Oct-Dec, 2021	4,147/760	AY.99.2	46.71%	57	11	0.525	0.376	0.309	0.248
Jan-Mar, 2022	1,948/140	BA.1	30.71%	21	37.5	0.596	0.487	0.339	0.294
Apr-Jun, 2022	88/30	BA.2	46.67%	10	33	0.713	0.636	0.395	0.358
Jul-Sep, 2022	106/32	BA.5.1.25	25.00%	11	34	0.716	0.676	0.400	0.379
Oct-Dec, 2022	17/5	DJ.1.1.1	60.00%	2	38	0.774	0.770	0.436	0.434
Jan-Mar, 2023	49/24	XBB.1.9	29.17%	12	40.5	0.885	0.790	0.511	0.448
Apr-Jun, 2023	76/38	FE.1.2	23.68%	17	42.5	0.922	0.861	0.548	0.494
Jul-Sep, 2023	/	/	/	/	/	/	/	/	/
Oct-Dec, 2023	/	/	/	/	/	/	/	/	/
Jan-Mar, 2024	/	/	/	/	/	/	/	/	/
Apr-May, 2024	/	/	/	/	/	/	/	/	/

* Dominant Percentage: (num of dominant lineage)/(num of variant);

Mean Mutations per variant sequence: MMut; Maximum Fitness: MaxFit; Mean Fitness: MFit;

Maximum Immune Escape Index: MaxIEI; Mean Immune Escape Index: MIEI

Table S8 Null model KS test result - Comparison of mean Fitness between real and random S protein sequences

Group	Test Type	Real Sequence Mean Fitness	Random Sequence Mean Fitness	KS Statistic	p-value	Conclusion
Global	Real vs. Random Mean KS test (160,892 vs 160,892)	0.3849	0.2046	0.6663	0.0000	Significant difference
Africa	Real vs. Random Mean KS test （2,382 vs 2,382）	0.2821	0.2048	0.5365	0.0000	Significant difference
Asia	Real vs. Random Mean KS test （6,904 vs 6,904）	0.4370	0.2047	0.7675	0.0000	Significant difference
Europe	Real vs. Random Mean KS test （3,788 vs 3,788）	0.3392	0.2049	0.6849	0.0000	Significant difference
North America	Real vs. Random Mean KS test (144,248 vs 144,248)	0.3883	0.2047	0.6675	0.0000	Significant difference
Oceania	Real vs. Random Mean KS test （966 vs 966）	0.2187	0.2041	0.3451	0.0000	Significant difference
South America	Real vs. Random Mean KS test （2,604 vs 2,604）	0.2831	0.2034	0.7065	0.0000	Significant difference

Note: The Kolmogorov-Smirnov (KS) test compares the fitness distribution of the mean of real sequences with that of random sequences. A p-value < 0.05 indicates a significant difference, suggesting that the empirical sequences deviate from the expectations of neutral evolution.

Table S9 Null model KS test result - Comparison of mean IEI between real and random S protein sequences

Group	Test Type	Real Sequence Mean Fitness	Random Sequence Mean Fitness	KS Statistic	p-value	Conclusion
Global	Real vs. Random Mean KS test (160,892 vs 160,892)	0.2894	0.1895	0.5647	0.0000	Significant difference
Africa	Real vs. Random Mean KS test （2,382 vs 2,382）	0.2235	0.1895	0.4434	0.0000	Significant difference
Asia	Real vs. Random Mean KS test （6,904 vs 6,904）	0.3253	0.1894	0.6360	0.0000	Significant difference
Europe	Real vs. Random Mean KS test （3,788 vs 3,788）	0.2554	0.1990	0.5805	0.0000	Significant difference
North America	Real vs. Random Mean KS test (44,248 vs 144,248)	0.2619	0.1896	0.5636	0.0000	Significant difference
Oceania	Real vs. Random Mean KS test （966 vs 966）	0.1951	0.1899	0.3345	0.0000	Significant difference
South America	Real vs. Random Mean KS test （2,604 vs 2,604）	0.2251	0.1881	0.6133	0.0000	Significant difference

Note: The Kolmogorov-Smirnov (KS) test compares the IEI value distribution of the mean of real sequences with that of random sequences. A p-value < 0.05 indicates a significant difference, suggesting that the empirical sequences deviate from the expectations of neutral evolution.

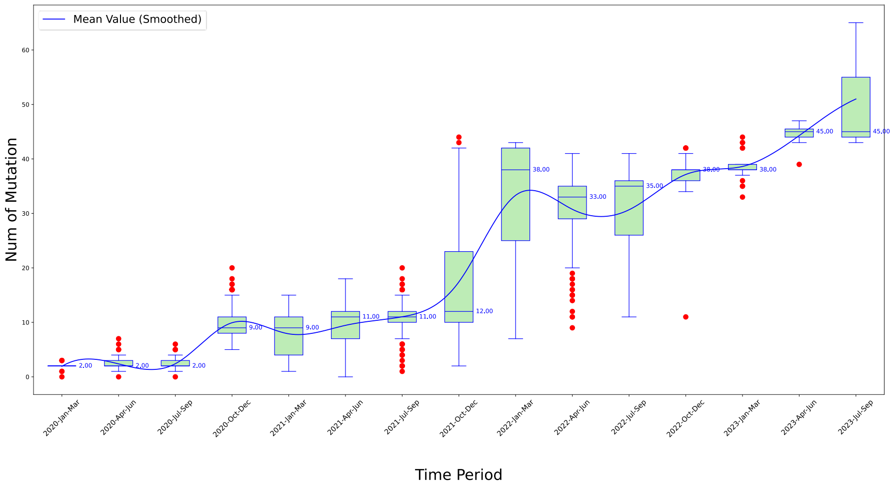

Figure S1 Temporal analysis of mutational frequency per variant sequence in Africa from 2020 to 2023

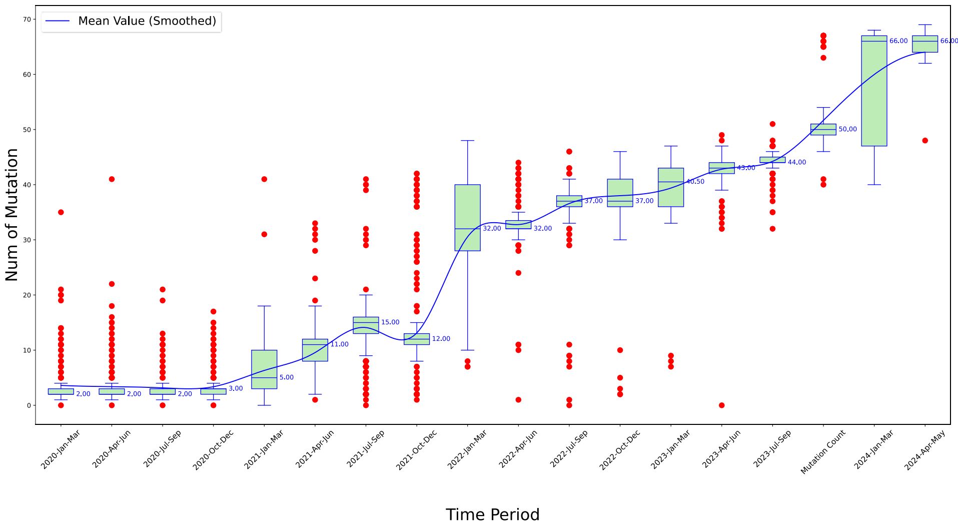

Figure S2 Temporal analysis of mutational frequency per variant sequence in Asia from 2020 to 2024

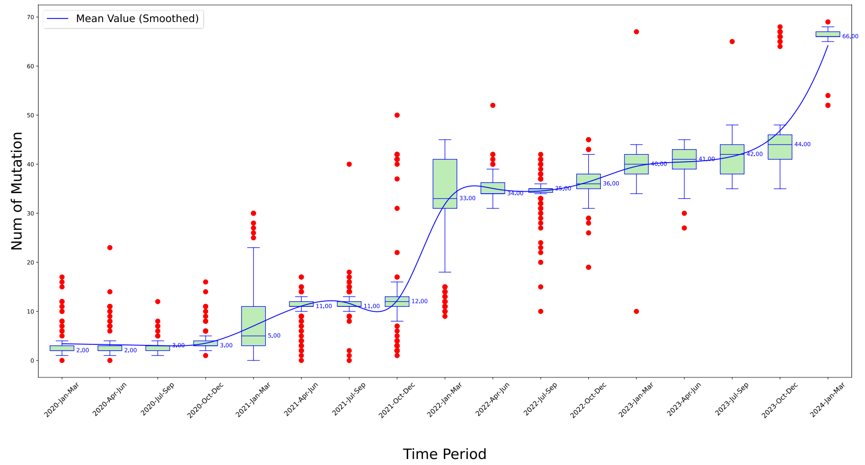

Figure S3 Temporal analysis of mutational frequency per variant sequence in Europe from 2020 to 2024

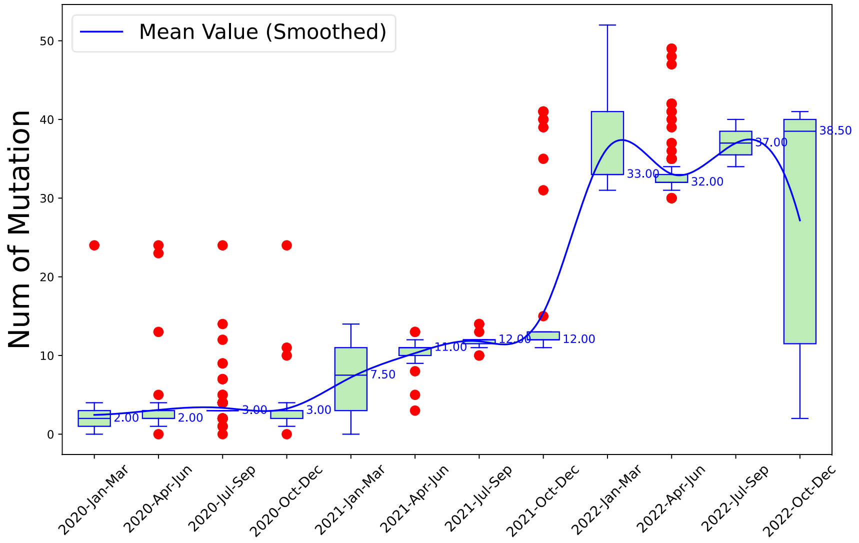

Figure S4 Temporal analysis of mutational frequency per variant sequence in Oceania from 2020 to 2022

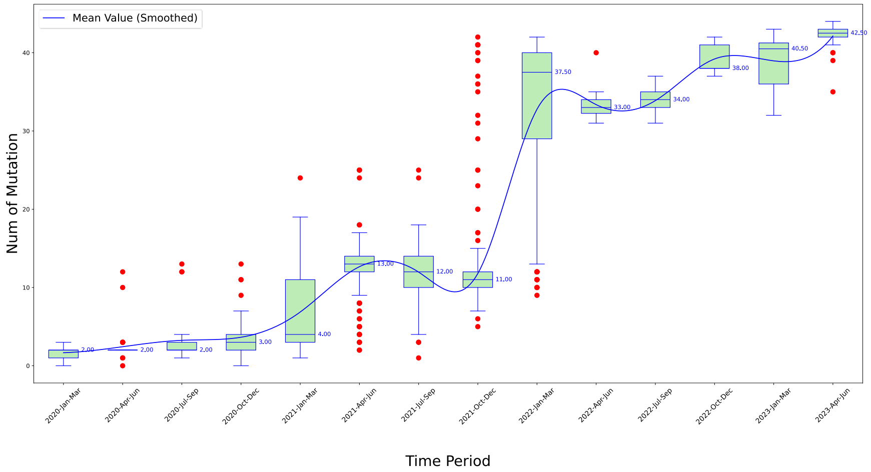

Figure S5 Temporal analysis of mutational frequency per variant sequence in South America from 2020 to 2022

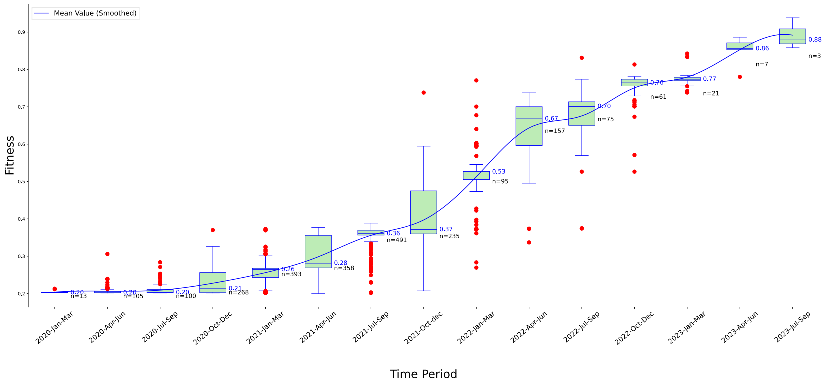

Figure S6 Temporal analysis of Fitness levels for S protein variants in Africa over various time periods

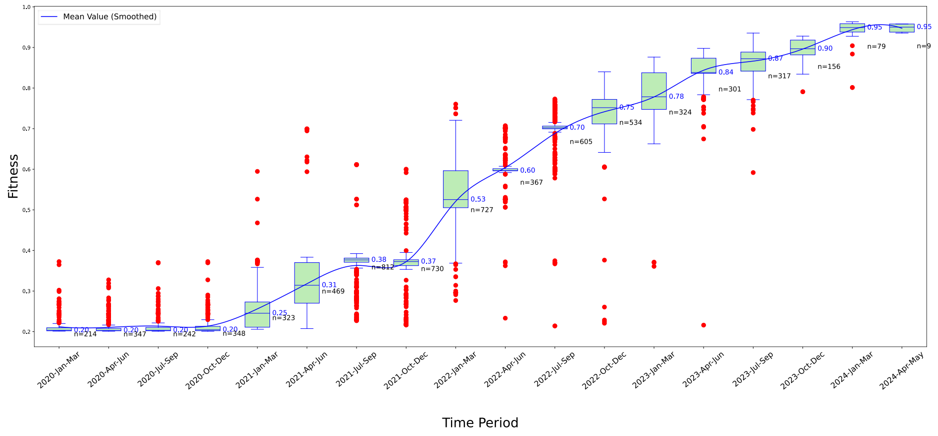

Figure S7 Temporal analysis of Fitness levels for S protein variants in Asia over various time periods

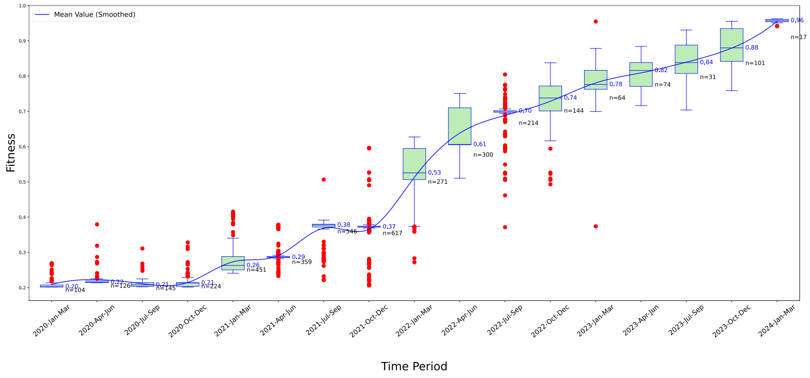

Figure S8 Temporal analysis of Fitness levels for S protein variants in Europe over various time periods

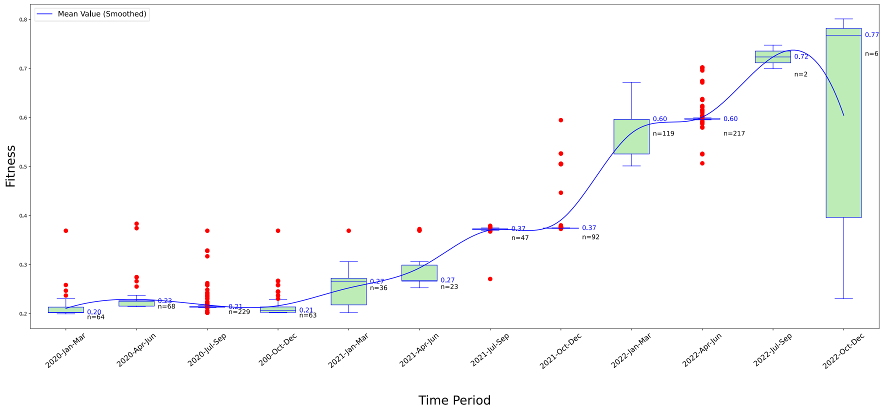

Figure S9 Temporal analysis of Fitness levels for S protein variants in Oceania over various time periods

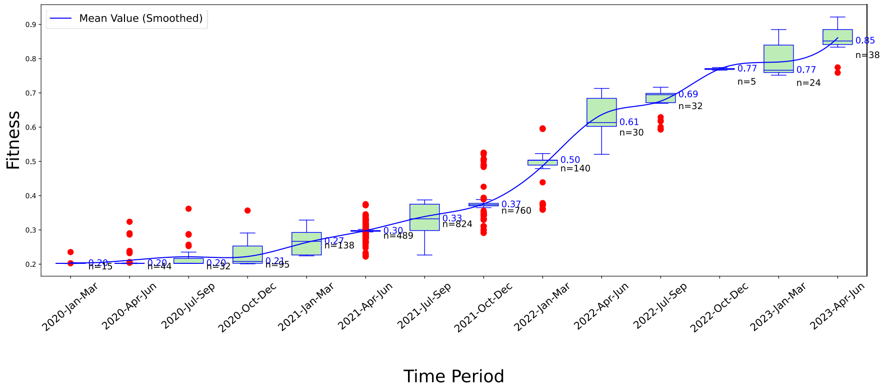

Figure S10 Temporal analysis of Fitness levels for S protein variants in South America over various time periods

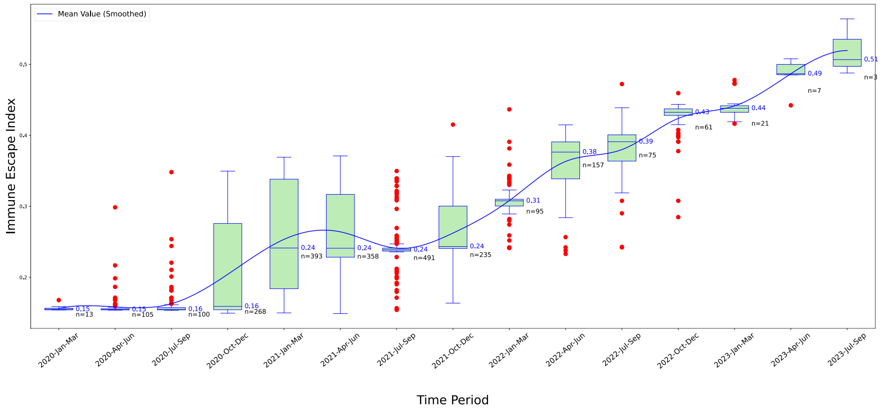

Figure S11 Temporal analysis of Immune Escape Index for S protein variants in Africa

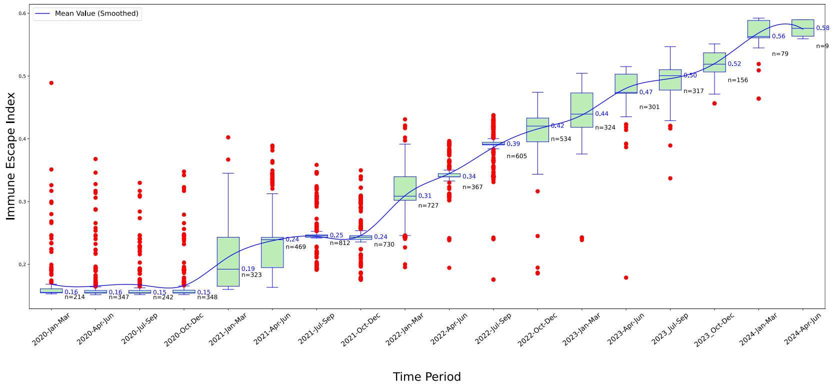

Figure S12 Temporal analysis of Immune Escape Index for S protein variants in Asia

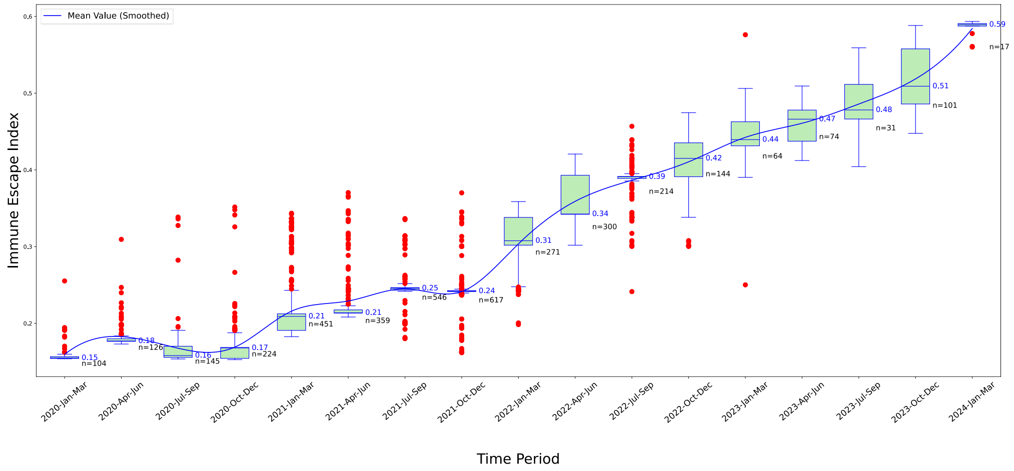

Figure S13 Temporal analysis of Immune Escape Index for S protein variants in Europe

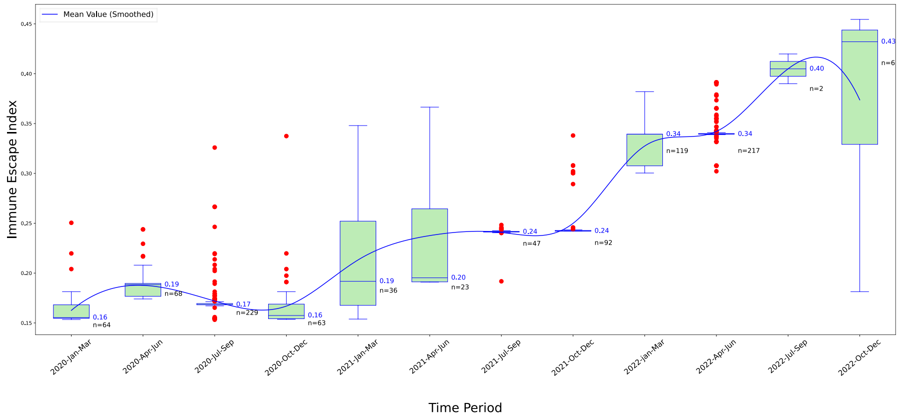

Figure S14 Temporal analysis of Immune Escape Index for S protein variants in Oceania

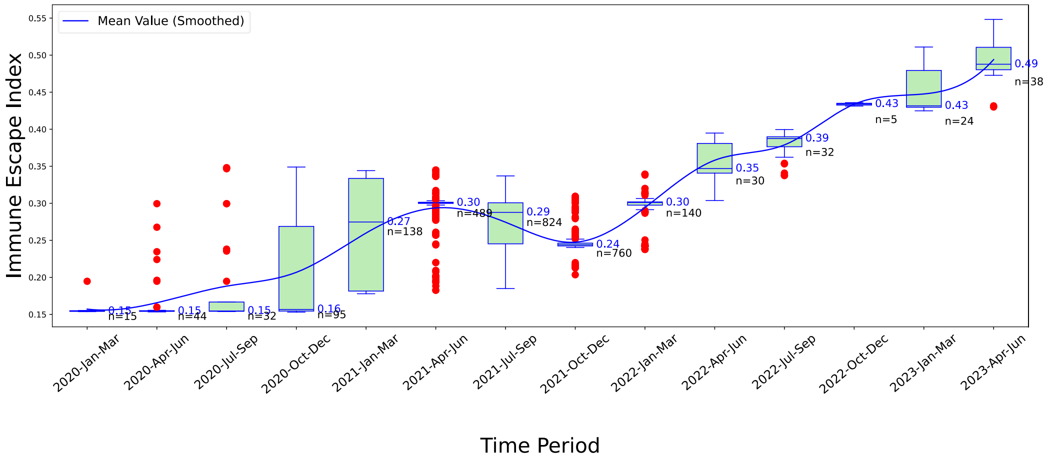

Figure S15 Temporal analysis of Immune Escape Index for S protein variants in South America
